# Dengue Dilemma in Nepal

**DOI:** 10.3126/nje.v12i4.50764

**Published:** 2022-12-31

**Authors:** Indrajit Banerjee, Jared Robinson, Brijesh Sathian

**Affiliations:** 1, 2 Sir Seewoosagur Ramgoolam Medical College, Belle Rive, Mauritius; 3 Geriatrics and long term care Department, Rumailah Hospital, Hamad Medical Corporation, Doha, Qatar

## Background

Dengue is an arthropod borne viral disease caused by the flavivirus DENV, which is transmitted via the mosquito vectors Aedes. aegypti and Aedes. Alboptictus. The dengue virus consists of four serotypes DENV1-4 [1]. According to the latest reports released by the (WHO) World Health Organization, the tropical disease dengue fever is reported to be endemic to 128 countries globally and poses a risk and threat to 3.6 billion people. Over 350 million people are estimated to be infected with the flavivirus annually [2]. Dengue is most commonly a tropical disease and thrives in warmer climatic conditions however, is endemic in subtropical and colder climates. Nepal is one such country in which Dengue is endemic, and since the 28th of September has experienced an upsurge and outbreak in cases [3].

### Nepalese outbreak

The first case of Dengue in Nepal was reported in 2004, after a traveller returned from India. Dengue has since then been an endemic disease within the country with all 4 serotypes (DENV-1,DENV-2,DENV-3 and DENV-4) being known to exist and circulate within the region. Based on epidemiological records the DENV-1 and DENV-2 serotypes have traditionally been the most incriminated serovars in terms of national disease burden according to the Epidemiology and the disease control division (EDCD). Since the beginning of January 2022 up until the 28th of September 2022 over 28000 cases of confirmed and suspected cases of dengue fever have been reported to the WHO with a total of 38 deaths being documented across all of Nepal’s provinces. To date the predominant implicated serotype or serotypes have not been identified. The province most adversely affected is the Bagmati province which accounts for 78.2% of the cases and 68.4% of the fatalities noted in Nepal. A further rise in cases has been noted as of the 8th of August 2022. This dengue outbreak is the largest recorded outbreak within the small mountainous regions recent history[3].

### Latest update

**Figure 1:**
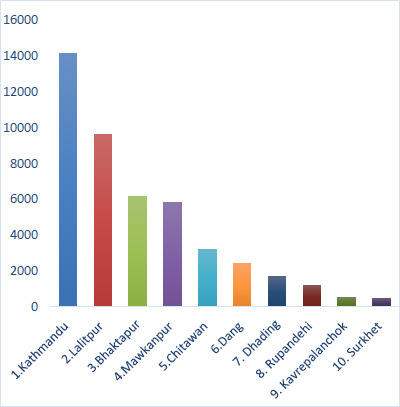
Dengue reporting: Top Ten Districts of Nepal

The latest data released by the Ministry of health and population of Nepal (11th December 2022), has recorded a total of 54232 cases with the Bagmati province accounting for 42143 thereof and Lumbini province 4984 respectively. To date a total of 67 deaths have been confirmed. The top 10 reporting districts are namely: 1.Kathmandu (14165) 2.Lalitpur (9614) 3.Bhaktapur(6145) 4.Mawkanpur (5830) 5.Chitawan (3188) 6.Dang (2405) 7. Dhading(1678) 8. Rupandehi (1203) 9. Kavrepalanchok (536) and 10. Surkhet (470) cases respectively which has been depicted in figure 1. The government and relative stakeholders are expecting a rise in cases and are thus intimating various strategies to curb the spread of the virus. Updates from the region are slow as the diagnosis and confirmation of suspected cases are not always direct and straightforward, secondly superadded to this the rough terrain and lack of access to medical facilities makes estimating the correct statistics and the timeously reporting thereof challenging [4,5].

### Nepalese response to the outbreak

The Nepalese government in partnership with various NGOs and the WHO have and are in the process of implementing numerous remedial and intervention measures to curb and control the current outbreak of Dengue sweeping through the country. To date some of the following measures and actions have been implemented.

- Virtual interactions and communication is ongoing between the EDCD and the various provinces, with coordination and responses taking place at both a local and district level in order to curb the spread of the viral spread.
- A budget of 139 palikas has been allocated across the 7 provinces for “search and destroy” in order to better curb the spread of the virus.
- The EDCD is in communication with both the schooling and hotel stakeholders in order to prepare the sectors for the ongoing outbreak.
- In the latter part of August the Lalitpur and Kathmandu districts initiated a mass scale search and destroy program.
- Technical orientation and sensitization programs for medical officers and physicians have been implemented in order to better prepare the healthcare system for improved and enhanced case management.
- The EDCD has circulated long lasting insecticidal mosquito nets to high case reporting hospitals in the Kathmandu region in order to prevent further viral transmission.
- Vectors surveillance is ongoing and most notably taking place in the districts of Kathmandu, Lalitpur and Dang [4].

### WHO risk assessment

The WHO has assessed the current outbreak figures, Nepal’s expertise, track record and abilities to handle such outbreaks. The WHO has classed the current outbreak as high risk on a National level, based on the fact that the disease is recurrent in Nepal and that re-infections occur. The level of severe Dengue cases has risen and therefore is cause for concern for the populations safety. The outbreak of Dengue has further placed increasing pressure on the already strained healthcare system as the outbreak of Dengue is coinciding with the rise of other vector borne disease such as Japanese Encephalitis and Scrub typhus. Climate change and altered breeding habits of the Aedes mosquitoes is increasing the likelihood of the disease to spread to a further extent and to higher levels which could result in a great propensity for epidemic potential. All of the above and further concomitant factors have thus instigated the high level of risk grading by the WHO and has thus intern warranted swift actions by the relative authorities [3,6].

### Treatment

No specific treatment or cure exists for Dengue fever, however the mainstay management consists of symptomatic treatment, fluid therapy and complication management. As an example pyrexia is managed with paracetamol and bleeding tendencies with judicious fluid therapy and blood component therapy if indicated. The CYD-TDV Dengue vaccine was licensed in December 2015. It is compatible for individuals aged 9 to 45 years in age. It is a live recombinant vaccine and is given in a 3 dose regimen. It is currently licensed in twenty countries internationally [7,8,9].

## Conclusion

Due to the treatment of the disease being predominantly supportive, the prevention of the spread through enhanced and improved vector control and elimination is recommended. The use of eradication programs, impregnated mosquito nets, larvicidal and chromosomal manipulation therapies are the most effective methods to aid and abet the control of this deadly virus. The coming months and response of the spread of the virus to the intimated control programs implemented in Nepal will act as a yardstick for the future development and course of the outbreak of Dengue within the mountainous region.
